# Simulation-based feedback systems in cardiopulmonary resuscitation training: a scoping review

**DOI:** 10.1186/s12873-026-01553-4

**Published:** 2026-04-06

**Authors:** Mahdieh Sabery, Zahra Batooli, Ebrahim Sabbarifard, Faezeh Ghaffari

**Affiliations:** 1 Trauma Nursing Research Center, Kashan University of Medical Sciences, Kashan, Iran; 2 Social Determinants of Health (SDH) Research Center, Kashan University of Medical Sciences, Kashan, Iran; 3 Department of Nursing, Kashan Branch, Islamic Azad University, Kashan, Iran; 4 Health Information Management Research Center, Kashan University of Medical Sciences, Kashan, Iran

**Keywords:** Cardiopulmonary resuscitation, Simulation, Feedback device, Education

## Abstract

**Background:**

Sudden cardiac arrest is among the leading causes of death worldwide and continues to be a public health issue. The quality of cardiopulmonary resuscitation (CPR) which relates to the depth, rate, and full recoil of chest compressions, is critical to return of spontaneous circulation (ROSC) and survival. Simulation-based environments provide a safe, controlled, and no-risk setting for practice of CPR, thus making it a prime setting to utilize the evaluation of feedback devices. This study sought to examine the influence of feedback systems on CPR quality in those simulations.

**Methods:**

A search was conducted in three major databases: PubMed, Web of Science, and Scopus. Search terms were tailored to the characteristics of each database and included a combination of keywords related to CPR and feedback devices. Inclusion criteria comprised English-language studies with experimental, quasi-experimental, or randomized controlled trial (RCT) designs that examined the effect of feedback devices on CPR quality in simulated scenarios and reported at least one CPR quality parameter.

**Results:**

The findings of this review of 31 studies published from 2006 to 2022 demonstrate that real-time feedback systems (including visual, auditory, combined, mobile, smartwatches, gamification, augmented/virtual reality, and video-based systems) produced an overall improvement in CPR quality with respect to compression depth and rate, full chest recoil, and correct hand positioning. Devices like TrueCPR, Mini-VREM, and smartwatch-based technology improved technical performance the most, while some accelerometer-based or AED integrated devices improved certain components, but produced decreased compression depth. Gamification worked well for younger learners, while combining video-based feedback with verbal feedback produced the best learning and retention of skills. On the contrary, some feedback tools increased the workload or contributed to distractibility, but some had a positive impact depending on the conditions of use. All in all, the evidence supported the use of real-time feedback in CPR quality in simulated environments.

**Conclusion:**

Real-time feedback is a valuable education, training, and clinical strategy for enhancing CPR skills. However, there needs to be a consideration between the ideal technology selected for the demographic population, educational or clinical setting, and the learning objective(s) intended. More research with a better methodology is warranted to examine the sustained impact of the technology, as well as the impact on the clinical outcomes of the patients involved.

## Introduction

Sudden cardiac arrest is one of the leading causes of global rates of mortality and poses a constant threat to the health of populations [1]. The American Heart Association (AHA) reports that survival to hospital discharge after experiencing out-of-hospital cardiac arrest and emergency resuscitation is only around 9–10% [2]. This relatively poor figure highlights a large barrier to the effectiveness of cardiopulmonary resuscitation (CPR). CPR is known to be the leading lifesaver for instances of cardiac arrest, yet it largely depends upon timely and accurate application.

The quality of cardiopulmonary resuscitation (CPR)—particularly in terms of important parameters such as chest compression depth, compression rate, and full recoil—plays a significant role in achieving return of spontaneous circulation (ROSC) as well as improving case survival rates [3]. However, studies demonstrate that even those with training can find it problematic to achieve high-quality CPR in real-world cases wherein they can be under physical pressure or under psychological stress. Factors like fatigue, anxiety, and coordination problems are typically cited as overriding causes of deterioration in performance for the critical procedure [4, 5].

To improve the quality of CPR and reduce human error, real-time devices that provide immediate feedback have been engineered. Such devices provide real-time readings of performance parameters—chest compression depth, rate, and recoil—allowing for real-time correction and making CPR performance much closer to the international standard [6, 7]. It has been demonstrated that use of such technologies can significantly optimize the quality of CPR [8–10]. Use of the devices has even been recommended by the American Heart Association in its 2015 and 2020 guidelines for use in teaching and to optimize resuscitation quality [11].

Since simulation-based environments are risk-free, controlled, and safe practice environments, they are considered the ultimate practice field and the superior test material for assessing the effectiveness of devices that provide feedback during CPR. Thus, this review aims to quantify the influence of feedback devices during CPR under simulated conditions. 

**Fig. 1:**
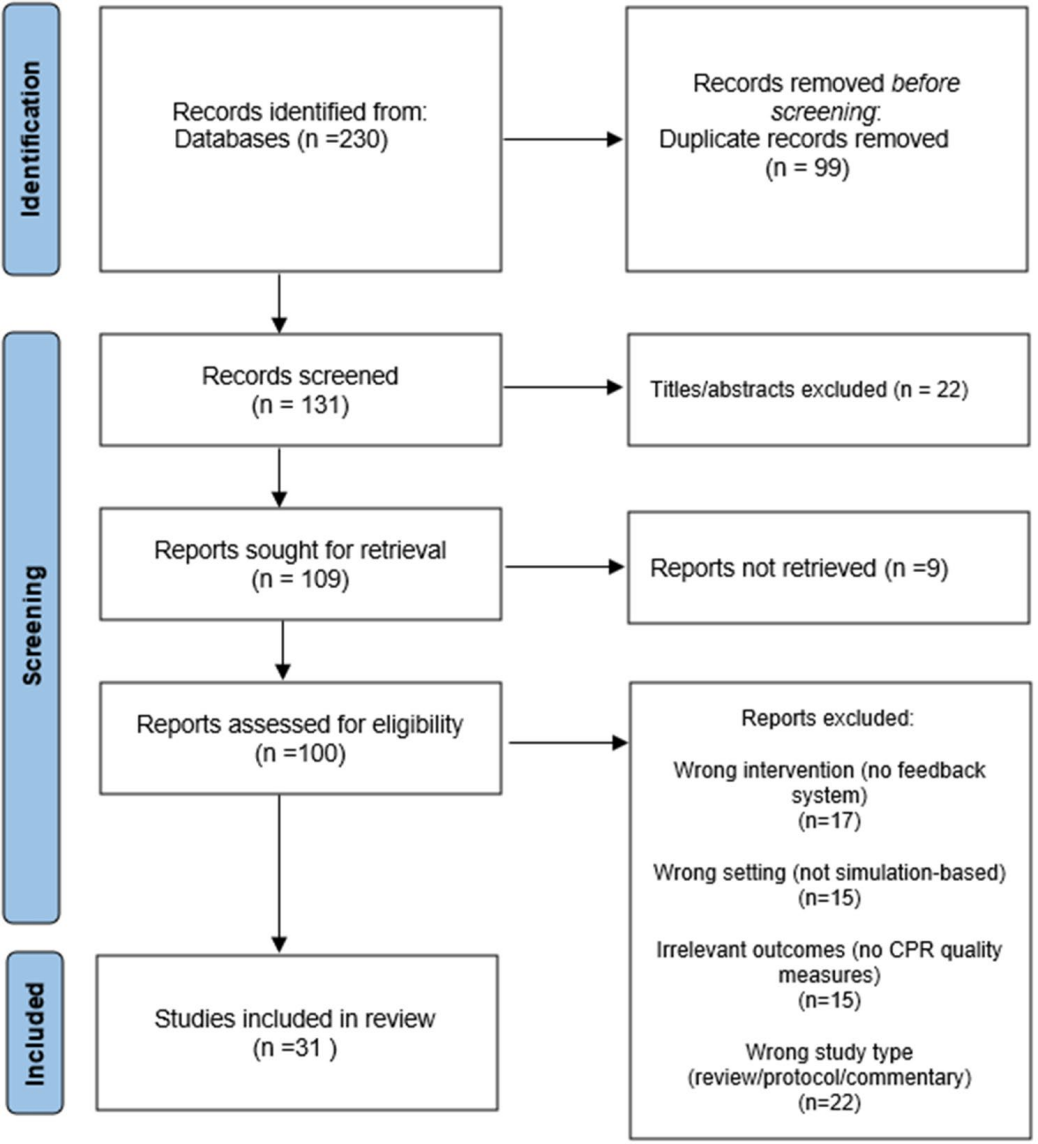
PRISMA diagram of study flow

## Methods

This study is a systematic review of existing evidence on the effectiveness of feedback devices in CPR training, with the specific aim of assessing their impact on CPR quality in simulation-based settings. The review was conducted in accordance with PRISMA (Preferred Reporting Items for Systematic Reviews and Meta-Analyses) guidelines to ensure transparency and comprehensive reporting (see Fig. 1). 

### Search strategy

A systematic search was carried out in three major databases—PubMed, Web of Science, and Scopus—on December 19, 2024. Search terms were tailored to the specifications of each database and included combinations of keywords related to cardiopulmonary resuscitation (e.g., *“Cardiopulmonary Resuscitation*,*” “CPR*,*” “cardiac arrest”*), feedback devices (e.g., *“audiovisual feedback*,*” “real-time feedback*,*” “sensory feedback”*), and simulation methods (e.g., *“simulation*,*” “virtual reality*,*” “augmented reality”*). Boolean operators (AND/OR) were used to maximize both accuracy and comprehensiveness. To further ensure inclusion of relevant studies, MeSH terms were also applied. The detailed search formulas are presented in Table 1.

**Table 1:** search strategy

Database	Query
WoS	“CardioPulmonary Resuscitation*” OR CPR OR “Cardio-Pulmonary Resuscitation*” OR “Cardio Pulmonary Resuscitation*” OR “Mouth-to-Mouth Resuscitation*” OR “Mouth to Mouth Resuscitation*” OR “Mouth-to-Mouth Resuscitation*” OR “cardiopulmonary resuscitation*” OR “resuscitation cardiopulmonary” OR “heart resuscitation*” OR “cardiac resuscitation*” OR ACLS OR BCLS OR “Basic Cardiac Life Support” OR “Advanced Cardiac Life Support” OR “cardiac arrest*” OR “heart arrest*” OR Asystole* OR “Cardiopulmonary Arrest” (Topic) and simulation* OR simulator* OR imitation* OR “virtual realit*” OR “Augmented Realit*” OR “Computerized Model*” OR “Computer Model*” OR “Computational Model*” OR “box train*” OR “box-trained” (Topic) and (Sensory NEAR/3 Feedback*) OR (Sensorimotor NEAR/3 Feedback*) OR (Audio NEAR/3 Feedback*) OR (Visual NEAR/3 Feedback*) OR (video NEAR/3 feedback*) OR (videotape NEAR/3 feedback*) OR “audio-visual feedback*” OR “audio visual feedback*” OR “audio/video-feedback*” OR “audio/video feedback*” OR “audiovisual feedback*” (Topic)
Scopus	TITLE-ABS-KEY ((sensory W/3 feedback*) OR (sensorimotor W/3 feedback*) OR (audio W/3 feedback*) OR (visual W/3 feedback*) OR (video W/3 feedback*) OR (videotape W/3 feedback*) OR “audio-visual feedback*” OR “audio visual feedback*” OR “audio/video-feedback*” OR “audio/video feedback*” OR “audiovisual feedback*”) AND TITLE-ABS-KEY (“CardioPulmonary Resuscitation*” OR cpr OR “Cardio-Pulmonary Resuscitation*” OR “Cardio Pulmonary Resuscitation*” OR “Mouth-to-Mouth Resuscitation*” OR “Mouth to Mouth Resuscitation*” OR “Mouth-to-Mouth Resuscitation*” OR “cardiopulmonary resuscitation*” OR “resuscitation cardiopulmonary” OR “heart resuscitation*” OR “cardiac resuscitation*” OR acls OR bcls OR “Basic Cardiac Life Support” OR “Advanced Cardiac Life Support” OR “cardiac arrest*” OR “heart arrest*” OR asystole* OR “Cardiopulmonary Arrest”) AND TITLE-ABS-KEY (simulation* OR simulator* OR imitation* OR “virtual realit*” OR “Augmented Realit*” OR “Computerized Model*” OR “Computer Model*” OR “Computational Model*” OR “box train*” OR “box-trained”)
PubMed	((((“audio-visual feedback*“[Title/Abstract] OR “audio visual feedback*“[Title/Abstract] OR “audio/video-feedback*“[Title/Abstract] OR “audio/video feedback*“[Title/Abstract] OR “audiovisual feedback*“[Title/Abstract]) OR (“Sensory Feedback“[Title/Abstract:~3] OR “Sensory Feedbacks“[Title/Abstract:~3] OR “Sensorimotor Feedback“[Title/Abstract:~3] OR “Sensorimotor Feedbacks“[Title/Abstract:~3] OR “Audio Feedback“[Title/Abstract:~3] OR “Audio Feedbacks” [Title/Abstract:~3] OR “Visual Feedback“[Title/Abstract:~3] OR “Visual Feedbacks“[Title/Abstract:~3] OR “video feedback” [Title/Abstract:~3] OR “video feedbacks” [Title/Abstract:~3] OR “videotape feedback” [Title/Abstract:~3] OR “videotape feedbacks” [Title/Abstract:~3])) OR (feedback, sensory[MeSH Terms])) AND ((simulation*[Title/Abstract] OR simulator*[Title/Abstract] OR imitation*[Title/Abstract] OR “virtual realit*“[Title/Abstract] OR “Augmented Realit*“[Title/Abstract] OR “Computerized Model*“[Title/Abstract] OR “Computer Model*“[Title/Abstract] OR “Computational Model*“[Title/Abstract] OR “box train*“[Title/Abstract] OR “box-trained“[Title/Abstract]) OR (((virtual reality[MeSH Terms]) OR (augmented reality[MeSH Terms])) OR (computer simulation[MeSH Terms])))) AND (((“CardioPulmonary Resuscitation*“[Title/Abstract] OR CPR[Title/Abstract] OR “Cardio-Pulmonary Resuscitation*“[Title/Abstract] OR “Cardio Pulmonary Resuscitation*“[Title/Abstract] OR “Mouth-to-Mouth Resuscitation*“[Title/Abstract] OR “Mouth to Mouth Resuscitation*“[Title/Abstract] OR “Mouth-to-Mouth Resuscitation*“[Title/Abstract] OR “cardiopulmonary resuscitation*“[Title/Abstract] OR “resuscitation cardiopulmonary“[Title/Abstract] OR “heart resuscitation*“[Title/Abstract] OR “cardiac resuscitation*“[Title/Abstract] OR ACLS[Title/Abstract] OR BCLS[Title/Abstract] OR “Basic Cardiac Life Support“[Title/Abstract] OR “Advanced Cardiac Life Support“[Title/Abstract] OR “cardiac arrest*“[Title/Abstract] OR “heart arrest*“[Title/Abstract] OR Asystole*[Title/Abstract] OR “Cardiopulmonary Arrest“[Title/Abstract]) OR (“Cardio Pulmonary Resuscitation“[Title/Abstract:~3] OR “Cardio Pulmonary Resuscitations“[Title/Abstract:~3])) OR (((cardiopulmonary resuscitation[MeSH Terms]) OR (heart arrest[MeSH Terms])) OR (advanced cardiac life support[MeSH Terms])))

### Inclusion and exclusion criteria

To be eligible for inclusion, studies had to meet the following criteria:

- Published in English.
- Investigated the impact of feedback devices (auditory, visual, audiovisual, or those based on smart technologies or augmented reality) on CPR quality in simulation-based scenarios.
- Reported at least one CPR quality parameter (compression depth, compression rate, full chest recoil, or hand position).
- Adopted an experimental, quasi-experimental, or randomized controlled trial (RCT) design.

Exclusion criteria were:

- Studies that did not provide quantitative data on CPR quality.
- Irrelevant articles, non-systematic reviews, or studies without full-text availability.

### Study selection and quality assessment

A total of 230 records were retrieved from the databases (PubMed: 52; Web of Science: 80; Scopus: 98). After removing 99 duplicates, 131 articles remained for title and abstract screening. During this phase, 22 studies were excluded for irrelevance, and 9 studies were excluded due to lack of full-text access.

The remaining 100 studies underwent quality appraisal using the Jadad scale (0–5). The Jadad scale was used as an initial screening tool to ensure minimum methodological quality, with a score ≥ 3 required for study inclusion. Of 100 studies, 69 studies scoring below 3 were excluded. The 31 articles with a score of 3 or higher were further evaluated using the CONSORT checklist (0–74). This tool was applied for quality screening and reporting appraisal, not as a formal risk-of-bias assessment. Although three studies scored below 37 (half of the maximum), they were retained due to the potential relevance of their data.

In total, 31 studies were included in the final analysis. Data extracted from these studies included author(s), year of publication, country, study objectives, study population, reported outcomes, and key findings.

### Data extraction and review process

Data extraction was carried out independently by two reviewers with the same data extraction form. If there was a disagreement between reviewers regarding the extraction of data or the assessment of quality, this was discussed, and a third reviewer was involved if necessary. To minimize bias and maximize reliability, each process and procedure, including screening, extraction, and assessment of quality, was done in duplicate and blinded.

### Data analysis

The extracted data were summarized in narrative form and tabulated for qualitative analysis, covering authorship, year, country, study objectives, participant population (students, healthcare professionals, or laypersons), outcomes, and main findings.

## Results

A total of 31 studies published between 2006 and 2022 were included in this review. These studies were conducted across several countries, including the United States, Canada, China, South Korea, Spain, and Austria. The largest number of studies originated from Canada, with peaks in publication during 2015 and 2019. The characteristics of the included studies are summarized in Table 2.

**Table 2:** Study characteristics

Num	Authors	year	Country	Objective	finding	Participant	Outcome
1	Savoldelli et al. [12]	2006	Canada.	-To investigate the value of the debriefing process during simulation and compare the educational efficacy of oral feedback and videotape-assisted oral feedback against a control (no debriefing).	- Participants who received oral feedback, with or without video assistance, showed significant improvement in nontechnical skills compared to those without debriefing. - There was no difference in improvement between oral and video-assisted oral feedback groups. - Exposure to a simulated crisis without debriefing offers little benefit, and valuable training can be achieved without video technology.	anesthesia residents	Valuable simulation training can be achieved even when video technology is not available.
2	Fischer et al. [13]	2011	Austria	To investigate if an AED with audiovisual feedback improves CPR parameters during standard BLS performed by trained laypersons.	- The use of an AED with audiovisual feedback improved CPR parameters such as compression rate, effective compressions, correct hand position, and reduced leaning. - However, the control group adhered better to the recommended compression depth, which is a key parameter for effective CPR. - Overall, the audiovisual feedback system improved some CPR-quality parameters but had a notable exception in decreased compression depth.	Flight attendants	An AED with audiovisual feedback improved some CPR quality parameters but decreased compression depth, a key parameter for cardiac output.
3	Chronister et al. [14]	2012	UAS	to evaluate the effect of two different debriefing styles on quality of student skills (assessment and psychomotor), skills response time, and knowledge retention in senior-level critical care students engaged in a cardiopulmonary arrest (CPA) simulation	ERPT scores significantly improved from CPA1 to CPA2 across all groups (*p* = 0.025) Group 1 (VA + V): Showed greater improvement from baseline, but not statistically significant (*p* = 0.71) Both groups completed all skills faster in CPA2 compared to CPA1 (*p* = 0.025) Group 1 (Video Assisted + Verbal) showed significantly faster performance in three key skills: Pulse check with initiation of CPR → *p* = 0.094 (marginal significance), Initial defibrillation shock → *p* = 0.042, Total time to resuscitation → *p* = 0.028 Group 2 (Verbal only): Slightly better improvement in VT recognition time, but not statistically significant A statistically significant difference between groups in knowledge retention → *p* = 0.008	Students in an undergraduate critical care	VA + V positively affects nursing skills and response times. Knowledge retention was more positively affected by V.
4	Buléon, et al. [15]	2013	France	To study the impact of the CPR meter feedback device on resuscitation performed by untrained rescuers	• The study compared chest compression (CC) quality between two groups: one with feedback guidance from a device (group G) and one without (group B). • Efficient Compression: Group G had a significantly higher rate of efficient compressions (71% vs. 26%). • Adequate CC Rate: Group G outperformed Group B (81% vs. 56%). • Average CC Depth, Peak Force, and Release Force: Improved in Group G, with less data dispersion around the mean. • Adequate CC Depth: Group G was superior (85% vs. 43%). • Learning Effects: No significant learning effect was observed between the two crossover phases.	Student	The use of the CPR meter significantly improved CC quality performed by students inexperienced in cardiopulmonary resuscitation
5	Semeraro et al. [16]	2013	Italy	To evaluates a new CPR feedback system (Mini VREM: Mini-Virtual Reality Enhanced Mannequin) designed to improve CC during training.	- The Mini-VREM system significantly improved chest compression performance by healthcare professionals and lay people in a simulated cardiac arrest scenario. - With Mini-VREM feedback, compressions were more adequate, and more compressions achieved the target rate and depth compared to performance without feedback. - The system was effective in improving compression rate and depth.	nurses and physicians (CPR experts) and engineers, students and researchers (non-CPR experts)	Motion detection technology can improve the quality of cardiopulmonary resuscitation (CPR) training.
6	Yeung et al. [17]	2014	UK	to compare the effect of three CPR prompt and feedback devices on quality of chest compressions amongst healthcare providers	- The pressure sensor device improved compression depth, while the accelerometer device decreased it, and the metronome had no effect. - All devices resulted in a decrease in compression rate. - The pressure sensor device reduced the proportion of compressions with inadequate depth.	Healthcare providers - Trained rescuers	A pressure sensor CPR feedback device improved compression depth, while an accelerometer device reduced it, and a metronome had no effect.
7	Song et al. [18]	2015	Korea	To evaluate the effectiveness of chest compression feedback during cardiopulmonary resuscitation in lateral tilted and semi recumbent positions.	The feedback device did not affect the quality of chest compressions in the supine position, but improved aspects of performance in the tilted positions. In the lateral tilted position, the median (IQR [range]) chest compression rate was 99 (99–100 [96–117]) compressions.min − 1 with and 115 (95–128 [77–164]) compressions.min − 1 without feedback (*p* = 0.05), and the proportion of compressions of correct depth was 55 (0–96 [0–100])% with and 1 (0–30 [0–100])% without feedback (*p* = 0.03). In the semi recumbent position, the proportion of compressions of correct depth was 21 (0–87 [0–100])% with and 1 (0–26 [0–100])% without feedback (*p* = 0.05)	- Basic life support-trained providers	feedback device improves the application of chest compressions during simulated cardiopulmonary resuscitation when the chest is tilted
8	Cheng et al. [19]	2015	Canada	To determine whether ‘just-in-time’ (JIT) CPR training with visual feedback (VisF) before CPA or real-time VisF during CPA improves the quality of chest compressions (CCs) during simulated CPA.	- The quality of CPR performed by healthcare professionals was initially poor, with low compliance rates for depth and rate of chest compressions. - JIT training and real-time visual feedback significantly improved compliance with American Heart Association guidelines for both depth and rate of chest compressions. - Combining JIT training and real-time VisF resulted in the highest compliance, although it was not significantly better than using either intervention alone.	CPR-certified health care professionals	Using novel and practical technology, JIT training before pediatric cardiopulmonary arrest or real-time visual feedback during pediatric cardiopulmonary arrest, alone or in combination, improves compliance with American Heart Association Guidelines for CPR that are associated with better outcomes.
9	Cheng et al. [20]	2015	Canada	To describe the degree of variability in the quality of CPR across 9 pediatric institutions, and determine if variability across sites would be affected by Just-in-Time CPR training and/or visual feedback during simulated cardiac arrest.	- The quality of CPR across multiple pediatric institutions is variable. - Significant variability in chest compression depth and rate was observed across institutions. - Variability in CPR quality persists even with the implementation of Just-in-Time training and visual feedback.	Healthcare Provider	There is significant variability in the quality of chest compressions provided during simulated cardiac arrest across pediatric institutions, even with interventions like real-time feedback and training.
10	Wutzler et al. [21]	2015	Germany	- Evaluate if chest compressions are more effective with the use of a novel feedback device compared to standard chest compressions. - Determine the absolute percentage of optimal chest compressions. - Determine the percentage of chest compressions in target rate. - Determine the percentage of chest compressions in target depth.	- The use of the novel feedback device increased the absolute percentage of optimal chest compressions from 27.9% to 47.6%. - There was a significant increase in the percentage of chest compressions in target depth (from 35.9% to 54.8%) and target rate (from 70.5% to 82.7%). - The feedback device significantly improved the quality of chest compressions in healthcare professionals.	healthcare professionals	A new audio-visual feedback device significantly improved the quality of chest compressions performed by healthcare professionals.
11	Davey et al.[22]	2015	New Zealand	to compare external chest compression data from the manikin-based Laerdal Skill Reporter (LSR) and the accelerometer-based Q-CPR technology, incorporated into the Philips MRx defibrillator, during CPR on a single Resusci Anne Simulator manikin.	- There was no significant difference in most chest compression quality metrics measured between the LSR and the Phillips Q-CPR devices. - Significant differences were found in the measurement of compression depth and duty cycle, with the Q-CPR device measuring lower values compared to the LSR.	Emergency medical specialist	The Laerdal Skill Reporter and Q-CPR devices show differences in measuring compression depth and duty cycle, but not other chest compression metrics.
12	Pavo et al. [23] .	2016	Austria	to investigate whether feedback from trained humans could be as effective for CPR quality as from a mechanical audio-visual feedback device in a two-rescuer scenario.	- Human feedback was equivalent to mechanical feedback in terms of CPR quality, as indicated by the effective compression ratio (ECR). - The human feedback group initiated chest compressions more quickly and had fewer incorrect decompressions compared to the mechanical feedback group. - Continuous feedback, whether human or mechanical, significantly improves CPR quality compared to no feedback.	medical students	Short structured feedback training is equivalent to a mechanical feedback device in two-rescuer basic life support.
13	González et al. [24]	2017	Spain	to analyze agreement in the assessment of external chest compressions (ECC) by 3 human raters and dedicated feedback software.	- The agreement among human raters and software in assessing ECC was generally low, with Cohen’s kappa coefficient and ICC being ≥ 0.54 in only a few instances and ≤ 0.45 in more than half. - Bland–Altman plots and survival–agreement plots showed significant dispersion and discordance in the data. - The study concludes that there is a significant lack of agreement among accredited raters and significant dispersion and inconsistency in data, questioning the reliability and validity of visual assessment of ECC.	volunteer health workers	Visual assessment of practical cardiopulmonary resuscitation skills by expert raters lacks reliability and validity.
14	Smereka et al. [25]	2017	USA	The aim of the study was to assess the role of the True CPR device in the process of teaching cardiopulmonary resuscitation in nursing students.	- The TrueCPR device improved chest compression rate, adequate chest compression rate, and full chest release percentage compared to standard BLS training. - Participants using TrueCPR had higher confidence in chest compression quality one month after training. - Overall, the TrueCPR device is associated with better resuscitation skills and confidence in performing CPR.	first year students of nursing	Using the True CPR device during CPR training leads to better chest compression skills and self-assessed confidence compared to standard CPR training.
15	Ahn et al. , [26]	2017	Korea	- To examine the effect of chest compression feedback via a smartwatch during cardiopulmonary resuscitation of manikins.	- The proportion of accurate chest compression depth was significantly higher in the intervention group using a smartwatch feedback system compared to the control group. - The mean compression depth and rate, as well as the proportion of complete chest decompressions, did not differ significantly between the two groups. - A smartwatch can assist rescuers by providing feedback on the ideal range for chest compression depth, aligning with American Heart Association guidelines.	medical students	Smartwatch feedback improves the accuracy of chest compression depth during simulated adult cardiac arrest.
16	Chen et al.[27]	2018	USA	- Step stool use is associated with improved compression depth regardless of provider height. - Increased provider height is associated with improved compression depth. - Visual feedback attenuates the effects of height and step stool use on compression depth.	- Primary objective: Explore whether step stool use is associated with improved CPR quality in providers of below-or above-average height. - Secondary objectives: - Explore whether adjusted height is associated with improved CPR quality. - Determine whether visual feedback or JIT training attenuates the effect of height on CPR quality. - Determine whether visual feedback or JIT training attenuates the effect of step stool use on CPR quality.	General population	Increased provider height is associated with improved compression depth, with visual feedback attenuating the effects of height and step stool use.
17	Abelairas-Gómez et al. [28]	2018	Spain	To analyse the acute muscular fatigue (AMF) in triceps brachii and rectus abdominis during compression-only and standard cardiopulmonary resuscitation (CPR) performed by certified basic life support providers.	- Compression-only CPR induces higher acute muscular fatigue than standard CPR. - Rectus abdominis muscle contraction time increased significantly during the fifth CPR period. - Participants with previous feedback training achieved better CPR quality results.	people with basic life support (BLS) current certification	Compression-only CPR induces higher acute muscle fatigue than standard CPR, and adequate rescuer strength is required to benefit from CPR quality feedback devices.
18	Heard et al. [29]	2019	USA	- To compare three methods of hands-only CPR education: classroom, kiosk, and video-only. - To assess the comparative effectiveness of these methods. - To explore the novel kiosk approach.	- The video-only group had lower total scores and compression depth scores compared to the classroom group. - There were no significant differences in total score between the classroom and kiosk groups, with the kiosk group excelling in hand position but weaker in compression depth. - The kiosk and classroom methods showed superior performance compared to the video-only method, with similar retention at 3-month follow-up.	General population	With regular retraining to prevent skills decay, the efficient and free hands-only CPR training kiosk has the potential to increase bystander intervention and improve survival from out-of-hospital cardiac arrest.
19	Otero-Agra et al. [30]	2019	Spain	To evaluate gamification methodology as compared with other existing methodologies when teaching cardiopulmonary resuscitation (CPR) to secondary school students.	- The gamification methodology resulted in significantly higher scores for CPR quality and correct rate compared to VFC and TC methods. - A higher proportion of students using gamification achieved a compression mean depth of over 50 mm compared to VFC and TC. - Gamification is considered an effective alternative teaching method for Basic Life Support (BLS) in younger individuals.	secondary school students	Gamification should be considered as an alternative teaching method for Basic Life Support (BLS) in younger individuals.
20	Wu et al. [31]	2019	China	To evaluate the quality of 2-minute continuous chest compressions performed by emergency staff and to determine the effect of a feedback system on maintaining the quality of these compressions.	- The proportion of optimal chest compressions was poor without feedback, remaining low throughout the 2-minute period. - The use of a feedback device significantly improved and maintained the quality of chest compressions from the first 30 s to the last 30 s. - The feedback device was helpful for maintaining the quality of chest compressions.	physicians and nurses	Use of the feedback device was helpful for maintaining the quality of continuous chest compressions.
21	Wagner et al [32]	2019	Canada	To test whether visual and verbal feedback compared with instructor-led feedback improve the quality of pediatric cardiopulmonary resuscitation (CPR).	- Chest compression performance significantly improved with both visual and verbal feedback compared to instructor-led feedback. - Improvements were noted in total compression score, correct hand position, and complete release, but not in compression depth. - Feedback devices should be implemented during pediatric resuscitation training to improve performance.	third-year medical students	Visual and verbal feedback improve the quality of pediatric cardiopulmonary resuscitation compared to instructor-led feedback.
22	Lu et al. [33]	2019	Taiwan	to investigate if a smartwatch with real-time feedback can improve CPR quality by healthcare professionals.	- The control group performed chest compressions at a significantly faster rate than recommended, while the intervention group maintained a rate closer to the guideline recommendation. - The intervention group achieved significantly deeper chest compressions compared to the control group. - The percentage of high-quality CPR was significantly higher in the intervention group, indicating improved performance with real-time feedback.	Emergency Department (ED) professionals	Using a smartwatch with real-time feedback improves the delivery of high-quality cardiopulmonary resuscitation by healthcare professionals.
23	Lin et al.[34] .	2019	China	- To compare the effectiveness of pretraining VAD with simulated errors (SE) versus trainees’ errors (TE) on BLS skills learning. - To assess whether SE can avoid drawbacks associated with using TE, such as threats to learners’ psychological safety.	- Pretraining video-assisted debriefing with simulated errors (SE) and trainees’ errors (TE) both significantly improved BLS skills scores compared to traditional training. - There was no significant difference in BLS skills scores between the SE and TE groups. - More trainees preferred SE for video-assisted debriefing due to psychological safety concerns.	third-year medical students	Pretraining video-assisted debriefing with simulated errors or trainees’ errors both improve basic life support skills learning in medical students.
24	Chan, et al. [35]	2019	Canada.	to determine whether an interactive mobile application providing audiovisual prompts, NRP Prompt, can help novice NRP providers learn the NRP algorithm more effectively and therefore improve their NRP performance.	- The NRP Prompt mobile application did not improve performance scores in simulated neonatal resuscitations immediately after training. - There were no significant differences in secondary outcomes such as times to positive-pressure ventilation, intubation, or chest compressions. - Potential reasons for the lack of improvement include distracting voice prompts and lack of customizability to user preferences.	second-year residents	A mobile application providing audiovisual prompts did not improve neonatal resuscitation performance in a simulation-based pilot study.
25	Dong et al.[36]	2020	China	- To determine the effect of the TCPRLink application on trained layperson T-CPR performance. - To evaluate the effectiveness of the TCPRLink application in a simulated cardiac arrest scenario. - To assess if the TCPRLink application improves CPR quality compared to conventional T-CPR instructions.	- The TCPRLink application improved T-CPR quality in trained laypersons by providing more effective chest compressions and reducing the need for counting the rhythm. - The TCPRLink group showed a higher chest compression rate compared to the T-CPR group, both initially and after 3 months. - Among participants aged 55–65 years, the TCPRLink group had a deeper chest compression depth compared to the T-CPR group.	adult participants (age 18–65 years) with T-CPR training experience	The TCPRLink application improved T-CPR quality in trained laypersons to provide more effective CCs and lighten the load of counting out the CC with the dispatcher in a simulated T-CPR scenario.
26	Dick-Smith et al. [37]	2020	Australia	- To investigate whether CPR feedback devices improve performance for nursing students. - To investigate the effects of three feedback modalities (visual: Laerdal SimPad; visual-embodied metaphoric: Innosonian Brayden Pro; and visual-audio: Physio-Control TrueCPR) on CPR performance in nursing students.	- All feedback modalities improved CPR performance compared to no feedback, with visual-audio feedback (TrueCPR) being the most effective. - Improved technical accuracy was observed in CPR skills such as chest compressions, ventilation, and cycle performance with feedback devices. - Real-time feedback devices significantly improved CPR psychomotor skills, demonstrating their immediate effectiveness.	Undergraduate Nursing Students	Real-time feedback modalities, especially visual-audio, improve CPR performance in nursing students compared to no feedback.
27	Leary et al [38]	2020	UAS	- To compare the effectiveness of an AR CPR training application (CPReality) with a standard AV feedback manikin in improving HCP CPR quality. - To determine if the AR technology can improve HCP CPR quality defined as chest compression rate and depth.	- The AR CPR training application did not improve CPR quality compared to the standard audio-visual feedback manikin in terms of chest compression rate and depth. - The AR system had slightly higher chest compression rates but lower depths compared to the standard manikin. - Both training methods resulted in low percentages of HCPs performing CPR within guideline recommendations, indicating a need for alternative training strategies.	Healthcare Provider	An AR CPR training application did not improve CPR quality compared to a standard CPR manikin training in healthcare providers
28	Lee et al. [39]	2021	Korea	To develop new audio call-to-video call transition protocols and test its efficacy and safety compared to conventional DACPR(C-DACPR).	Hand Positioning: • V-DACPR (Rapid vs. C-DACPR): 92.7% vs. 82.4% (*p* = 0.03) • V-DACPR (Delayed vs. C-DACPR): 91.1% vs. 82.4% (*p* = 0.07) • Overall V-DACPR vs. C-DACPR: 91.9% vs. 82.4% (*p* = 0.02) Chest Compression Depth: • V-DACPR (Rapid vs. C-DACPR): 40.7 mm vs. 35.9 mm (*p* = 0.01) • V-DACPR (Delayed vs. C-DACPR): 40.9 mm vs. 35.9 mm (*p* = 0.01) • Overall V-DACPR vs. C-DACPR: 40.8 mm vs. 35.9 mm (*p* < 0.01) Total No-Flow Time: • C-DACPR vs. V-DACPR: 22.6 s vs. 19.8 s (*p* = 0.33) • Mean Compression Depth Over Time: • C-DACPR: Decrease observed (*r* = − 0.22, *P* < 0.01) • V-DACPR (Rapid): No significant change (*r* = − 0.05, *p* = 0.52) • V-DACPR (Delayed): No significant change (*r* = − 0.12, *p* = 0.08) Adequate Hand Positioning Over Time: • V-DACPR (Rapid): Improvement observed (*r* = 0.25, *p* < 0.01) • V-DACPR (Delayed): Improvement observed (*r* = 0.19, *p* < 0.01) • C-DACPR: No significant change (*r* = − 0.01, *p* = 0.98)	Volunteers aged 18 years or older	Participants in the V-DACPR groups performed higher quality chest compression with higher appropriate hand positioning and deeper compression depth compared to the C-DACPR group
29	Lin et al. [40]	2021	China	To compare the effect of synchronous online and face-to-face cardiopulmonary resuscitation (CPR) training on chest compressions quality in a manikin model.	There was no statistically significant difference in chest compression (CC) quality between the TN and FN groups. No statistically significant differences were found between the TF and FF groups regarding correct hand position, CC depth, appropriate CC depth, complete chest recoil, or CC rate. The FF group demonstrated a significantly higher rate of appropriate CC compared to the TF group (*p* = 0.045). In the face-to-face training groups, participants using the AVF device showed significantly greater CC depth, appropriate CC depth, CC rate, and appropriate CC rate. There were no statistically significant differences in correct hand position (*p* = 0.191) and appropriate CC depth (*p* = 0.123). In the synchronous online training groups, the AVF device had minimal impact on CC rate (*p* = 0.851). Chest compression (CC) has Increased but the difference was not statistically significant (*p* = 0.178).	fourth-year medical students	Synchronous online training with AVF devices seems to be a suitable replacement for face-to-face training to offer adequate bystander CPR chest compression training.
30	Wagner et al. [41]	2022	Austria	To investigate the effect of feedback devices on visual attention and the quality of pediatric resuscitation.	- The quality of pediatric resuscitation significantly improved with real-time feedback. - The use of feedback devices shifted visual attention from the manikin to the device. - Feedback devices increased subjective workload, but this increase was small and did not interfere with resuscitation quality.		Feedback devices improve the quality of pediatric resuscitation but also increase the workload and shift visual attention away from the patient.
31	Jeffers et al. [42]	2022	USA	- To determine whether AR-CPR changes a user’s CC performance. - To gather feedback and suggestions for future development.	- The AR-CPR feedback system significantly improved chest compression performance, with 73% perfect epochs compared to 17% without it. - Subjects enjoyed using the AR-CPR system and felt it improved their performance. - The system showed significant performance change closer to CPR guidelines.		An augmented-reality cardiopulmonary resuscitation feedback system improved pediatric chest compression performance in a simulation-based setting.

The reviewed research examined a wide range of feedback systems, including auditory, visual, audiovisual feedback, mobile applications, smartwatches, gamification approaches, augmented/virtual reality tools, and video-based feedback. Most participants were medical students or healthcare professionals. The primary CPR quality parameters assessed were compression depth, compression rate, full chest recoil, and correct hand placement during chest compressions.

Most studies reported that real-time feedback significantly improved CPR quality, particularly in achieving the recommended compression rate and depth, correct hand placement, and complete chest recoil. Devices such as TrueCPR, Mini-VREM, and smartwatch-based systems consistently enhanced technical performance [37, 25, 43]. However, not all findings were positive: for example, some accelerometer-based or AED-integrated audiovisual feedback devices improved certain parameters but were associated with a reduction in compression depth, which is critical for effective cardiac output [13]. 

Gamification proved effective in improving CPR learning among younger students [30]. Smartwatch feedback increased accuracy in compression rate and depth [26, 33], while augmented or virtual reality tools yielded mixed results—some improved performance, whereas others performed worse compared to standard manikins. Mobile applications were beneficial in certain contexts, but at times acted as a source of distraction [43, 42].

In addition, combining verbal feedback with video-based feedback enhanced both skill acquisition and knowledge retention [12, 14, 34]. Feedback provided by trained instructors was, in some settings, found to be as effective as mechanical systems [23]. Moreover, feedback improved team coordination and reduced compression gaps, although it slightly increased workload and caused some visual attention to shift from the patient to the device [41]. Finally, human assessment of CPR quality showed poor agreement with software-based evaluations, raising concerns about its reliability [24].

In sum, evidence suggests that real-time feedback systems generally enhance both the quality of CPR and recommended compression characteristics in the simulation environment. However, the effectiveness of these devices also depends on the context and type of feedback system. Some systems may even intuitively worsen performance characteristics such as compression depth.

## Discussion

This review demonstrated that the use of feedback systems in CPR training within simulation environments generally enhances chest compression quality. The most notable improvements were observed in compression rate (from ~ 70% to > 82%) and adequate compression depth (from 35.9% to 54.8%) [21]. However, not all devices yielded positive results. For instance, Fischer et al. reported that an AED with audiovisual feedback reduced compression depth, a parameter critical for effective circulation [13]. Similarly, Leary et al. found that augmented reality did not produce significant improvements compared with standard manikins [38]. Collectively, the impact of these tools ranged from substantial benefits in novices to limited or even negative effects among experienced providers [37, 15, 17].

These findings reinforce what earlier studies have shown: real-time feedback plays a crucial role in improving CPR performance. That said, not all feedback tools are created equal. Some devices, for example, may lead to shallower chest compressions, highlighting differences in reliability. These variations often come down to the technology used—like pressure sensors versus accelerometers—as well as differences in participants’ experience and the specific training environment.

From a teaching perspective, tools like TrueCPR and Mini-VREM are great for quickly building CPR skills. But like any skill, if you don’t practice regularly, performance can slip over time. Research shows that short refresher sessions each month can help learners retain their skills. The American Heart Association also recommends booster sessions every 1 to 6 months after intensive training, which not only helps maintain proficiency but can, in some cases, even reduce mortality rates.

Using feedback systems in nursing and medical education has the potential to make CPR training more consistent, build learners’ confidence, and reduce the gap in performance between different institutions. But implementing these technologies isn’t always simple. Challenges like added mental load, possible distractions from patient care, and the cost of equipment need to be considered carefully. Institutions should weigh these factors thoughtfully when planning training programs or deciding whether to bring in new tools.

Although there is a growing body of evidence demonstrating the effectiveness of smart feedback devices in increasing immediate performance and improving learners’ proficiency, several important implications remain unexplored in the literature. Future studies could assess the long-term impact of these technologies on skill retention, transfer of learning to real-world clinical or operational settings, and sustained behavior change. In addition, issues such as cost-effectiveness, user acceptance, cognitive load, and standardization remain areas that require further investigation. Addressing these gaps through studies will be essential to fully understand the broader educational and practical value of smart feedback systems.

## Limitations

There are limitations to this review. First, the search was concluded on September 16, 2024, and more recent articles may not have been captured. Second, only circulations generated in English texts were included, which may have limited the evidence from non-English sources. Further reviews can consider broader inclusion of languages and include recent studies.

## Conclusion

In conclusion, real-time feedback systems remain a valuable pedagogical and clinical approach to improving the quality of CPR in a simulation-based study. However, its effectiveness is tempered by the type of device, the learning group, and the context of the training. Thus, a carefully selected technology, in combination with some degree of reinforcement (spaced and booster training), will assist in the retention of skill acquisition. There remains a need for more high-quality studies steering long-term engagement and transference to actual patient outcomes.

## Data Availability

All data used and analyzed during this study are included in this published article.
